# Description of a Rare Pyomelanin-Producing Carbapenem-Resistant Acinetobacter baumannii Strain Coharboring Chromosomal OXA-23 and NDM-1

**DOI:** 10.1128/spectrum.02144-22

**Published:** 2022-08-10

**Authors:** Feng Zhao, Haiyang Liu, Yue Yao, Linghong Zhang, Zhihui Zhou, Sebastian Leptihn, Yunsong Yu, Xiaoting Hua, Ying Fu

**Affiliations:** a Department of Clinical Laboratory, Sir Run Shaw Hospital, Zhejiang University School of Medicine, Hangzhou, Zhejiang, China; b Key Laboratory of Precision Medicine in Diagnosis and Monitoring Research of Zhejiang Province, Hangzhou, Zhejiang Province, China; c Department of Infectious Diseases, Sir Run Shaw Hospital, Zhejiang University School of Medicine, Hangzhou, Zhejiang, China; d Key Laboratory of Microbial Technology and Bioinformatics of Zhejiang Province, Hangzhou, Zhejiang, China; e Regional Medical Center for National Institute of Respiratory Diseases, Sir Run Run Shaw Hospital, Zhejiang University School of Medicine, Hangzhou, Zhejiang, China; f Zhejiang University-University of Edinburgh (ZJU-UoE) Institute, Zhejiang University, International Campus, Haining, Zhejiang, China; g College of Medicine & Veterinary Medicine, University of Edinburgh Medical School, Edinburgh, United Kingdom; Shenzhen Bay Laboratory

**Keywords:** CRAB, pyomelanin, *hmgA* overexpression, Tn*2006*, Tn*125*-like transposon

## Abstract

Carbapenem-resistant Acinetobacter baumannii (CRAB), which belonged to global clones 1 (GC1) or 2 (GC2), has been widely reported and become a global threat. However, non-GC1 and non-GC2 CRAB strains are not well-studied, especially for those with rare phenotype. Here, one pyomelanin-producing CRAB strain (A. baumannii DETAB-R21) was isolated from oral swab in the ICU. Antimicrobial susceptibility testing showed it was resistant to carbapenems, ceftazidime, levofloxacin, and ciprofloxacin. DETAB-R21 was ST164_Pas_ and ST1418_Oxf_ with KL47 and OCL5, respectively. Whole-genome sequencing (WGS) analysis revealed chromosome contained three copies of *bla*_OXA-23_ on three 4,805-bp Tn*2006* composite transposons with various novel 9-bp target site duplications (TSD). A Tn*125*-like structure, including *bla*_NDM-1_, a novel 4,343 bp composite transposon encoding *bla*_CARB-16,_ and three prophage regions were also identified. Importantly, *hmgA* was interrupted by a Tn*2006* and contributed to pyomelanin production and further confirmed by *hmgA* overexpression. Furthermore, A. baumannii irradiated with UV light, DETAB-R21 showed a higher relatively survival rate compared to a control strain that did not produce pyomelanin. No effects of pyomelanin were observed on disinfectants susceptibility, growth, or virulence. In conclusion, pyomelanin-producing CRAB carrying the *bla*_NDM-1_ and *bla*_OXA-23_ genes embedded in the bacterial chromosome is of grave concern for health care settings, highlighting the need for effective measures to prevent further dissemination.

**IMPORTANCE** Pyomelanin production is a quite rare phenotype in A. baumannii. Moreover, the mechanisms leading to the pyomelanin production was still unclear. Here, we for the first time, confirmed the mechanism of pyomelanin production, and further investigated the impact of pyomelanin on disinfectants susceptibility, growth, virulence, and UV irradiation. More importantly, many mobile genetic elements (MGEs), including three copies of Tn*2006* composite transposons, one copy of *bla*_NDM-1_ on the Tn*125*-like structure and three prophage regions, were identified in the chromosome, demonstrated strong plasticity of A. baumannii genome. Our study provides important insights into the new rare ST164_Pas_ A. baumannii strain with high level carbapenem resistance, which is of great threat for patients. These findings will provide important insights into the resistance gene transfer via transposition events and further spread in the clinic.

## INTRODUCTION

Acinetobacter baumannii is a major cause of nosocomial infections affecting mainly patients in the intensive care unit (1). Importantly, in 2019, Centers for Disease Control and Prevention (CDC) report carbapenem-resistant Acinetobacter as “Urgent Threats” (2). Nosocomial outbreaks causing carbapenem-resistant A. baumannii (CRAB) strains are emerging rapidly worldwide and pose a huge threat to global health (3), particularly due to the increasing frequency of multidrug resistant (MDR) infections (4).

Most CRAB infections are caused by strains that belong to global clones 1 (GC1) or 2 (GC2), with GC2 accounting for the vast majority of sequenced carbapenem-resistant isolates (5). However, non-GC1 and non-GC2 CRAB strains, such as ST164_Pas_, are reported rarely. As well documented, OXA-23, the class D β-lactamase to be identified from CRAB, still remains the most common contributor to carbapenem resistance (6). The *bla*_OXA-23_ gene is most frequently located in Tn*2006*, Tn*2008* or Tn*2009* transposons flanked by the insertion sequence IS*Aba1* and was widely reported (7). However, some metallo-β-lactamase genes (*bla*_NDM_, *bla*_VIM,_ and *bla*_IMP_) are relatively rare in A. baumannii but are occasionally found in the bacterial chromosome or in plasmids (5, 8). The *bla*_NDM-1_ gene is commonly located in the Tn*125* transposon (9). However, no studies have reported the existence of *bla*_OXA-23_ and *bla*_NDM-1_ in the chromosome of A. baumannii at the same time.

Pigmentation of clinical strains of CRAB has rarely been observed. The presence of a reddish-brown pigment, pyomelanin, causes a quite rare phenotype, reported only twice (10, 11). The overproduction of the pyomelanin is caused due to changes in the tyrosine metabolic pathway and is based on the deletion of the *hmgA* encoding homogentisate dioxygenase (HmgA) by IS*Aba1* (11). Pyomelanin-producing A. baumannii can show resistance to a wide range of antimicrobial drugs (11), which in turn could become a new challenge in the clinical setting, especially in the ICU.

Here, we characterize the genome of a rare pyomelanin-producing CRAB (DETAB-R21) collected from the oral swab from patient in the ICU, which carries three copies of *bla*_OXA-23_ in Tn*2006* and one copy of *bla*_NDM-1_ in Tn*125-*like composite transposons in the chromosome. To our knowledge, we confirmed the mechanism of pyomelanin generation for the first time while also investigating the fitness cost, virulence, and UV resistance of the pyomelanin-producing CRAB isolate.

## RESULTS

### Genome analysis of the clinical strain A. baumannii DETAB-R21.

The complete genome of A. baumannii DETAB-R21 was obtained from a hybrid assembly of Illumina and Nanopore (MinION) reads sequencing data. In addition to a chromosome of a size of 3,862,196 bp, the strain contains five plasmids (Table 1). The chromosome has an overall GC content of 39% (Table 1) and contains 20 XerC/XerD (C/D) and XerD/XerC (D/C) recombinases recognition sites (Fig. S1). Based on the Pasteur and Oxford MLST schemes, DETAB-R21 is ST164_Pas_ (*cpn60*-40, *fusA*-3, *gltA*-7, *pyrG*-2, *recA*-40, *rplB*-4, *rpoB*-4) and ST1418_Oxf_ (*cpn60*-36, *gdhB*-58, *gltA*-21, *gpi*-114, *gyrB*-48, *recA*-42, *rpoD*-4). Both MLST schemes of XH1935 are identical to DETAB-R21, with only a 5-bp difference in their genome based on the SNP analysis.

**TABLE 1:** Characteristics of DETAB-R21 genome components

Element	Replicon type	Size (bp)	GC content	Antibiotic resistance genes
Chromosome		3,862,196	38.97%	*bla*_ADC-52_, *bla*_ADC-176_, *bla*_OXA-91_, *bla*_OXA-23_, *bla*_NDM-1_, *ble*_MBL_, *bla*_CARB-16_, *aph(3′)-VI, ant(3′’)-IIa*
pDETABR21-1		12,709	32.94%	
pDETABR21-2		4,554	41.26%	
pDETABR21-3		2,924	37.82%	
pDETABR21-4	Aci1	2,742	37.24%	
pDETABR21-5	Aci4	2,309	38.54%	

The software Bautype showed DETAB-R21 contains OC locus 5 (OCL-5), matching the reference sequence with 99.64% nucleotide identity. The K locus in DETAB-R21 is KL47, to which it matches 100% of the locus with an overall nucleotide identity of 96.89%. Similarly, the software Kaptive revealed identical KL and OCL types.

**FIG 1:**
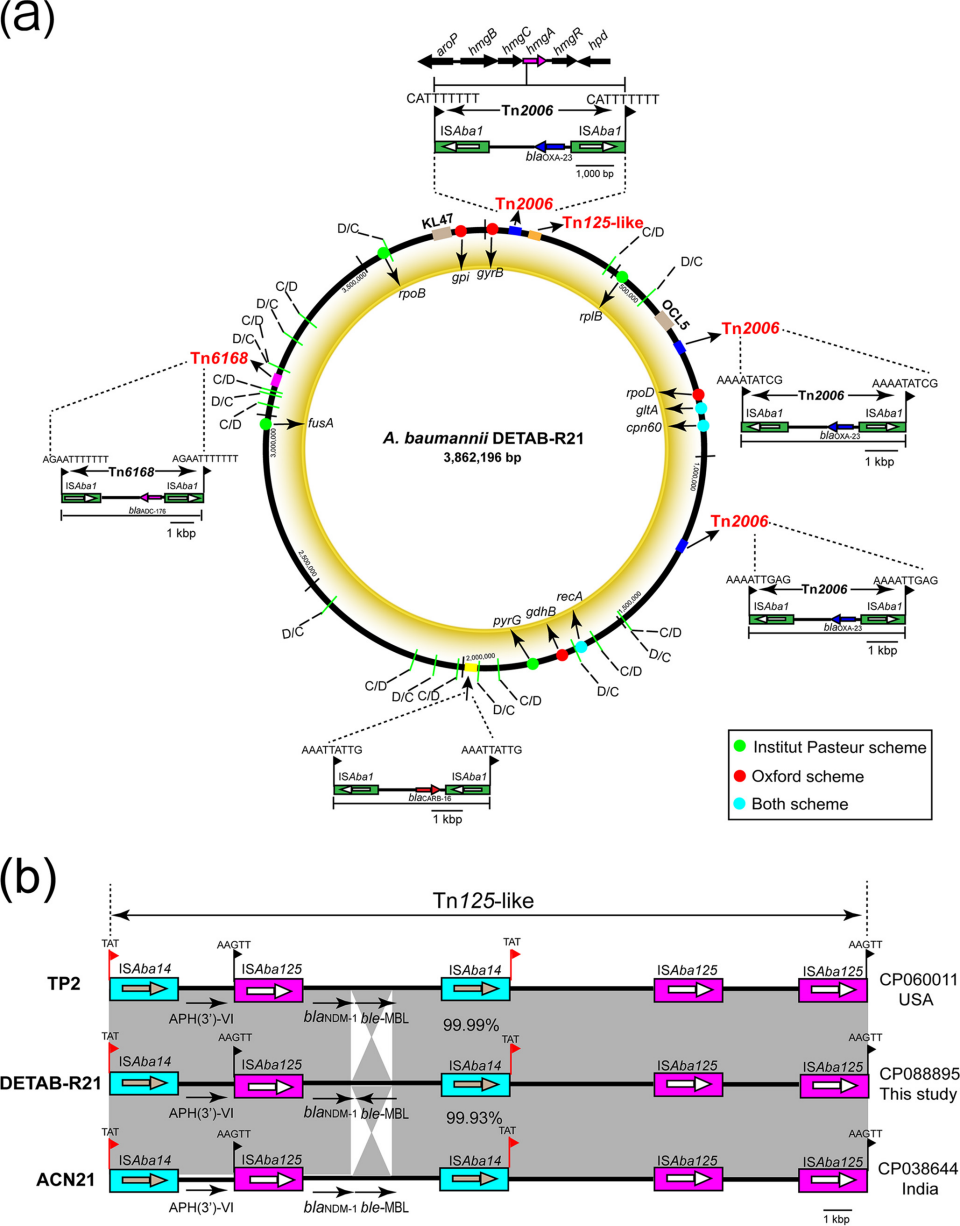
A. baumannii DETAB-R21 chromosome and transposons structure. (a) Circular map of the A. baumannii DETAB-R21 chromosome. Housekeeping genes used in MLST schemes are indicated by various colors. The structure of Tn*2006* is shown as blue filled box. *hmgA* was interrupted by one copy of Tn*2006.* Tn*125*-like is shown as orange filled boxes. Tn*6168* is shown as red violet filled box. The novel transposon of *bla*_CARB_ is shown as yellow filled box. Short black lines represent the p*dif* sites, with the orientations of XerC (C) and XerD (D). Target site duplications (TSD) shown on either side of the transposons are indicated using black flags. (b) Structure of Tn*125*-like compared with A. baumannii TP2 (CP060011) *and* A. baumannii ACN21 (CP038644). Horizontal arrows represent the direction of genes, with black arrows indicating resistance genes. Light blue filled boxes indicate IS*Aba14* copies. Red violet filled boxes indicate IS*Aba125* copies. Gray shades indicate regions with 99.9.%–100% identity. TSD is shown as flag using red or black.

ResFinder detected nine antibiotic resistance genes in the DETAB-R21 genome (Table 1). All resistance genes are in the chromosome (Table 1). Two of these, *bla*_ADC-52_ and *bla*_OXA-91_, represent the native AmpC and OXA-51 β-lactamase genes. Additionally, one Tn*6168* carried *bla*_ADC-176_ is located in the chromosome with 11-bp target site duplications (TSD) (Fig. 1a). Moreover, there are three copies of *bla*_OXA-23_ on the 4,805-bp Tn*2006* composite transposon structure with the different sequence of the 9-bp TSD on either side and one copy of *bla*_NDM-1_ and *ble*_MBL_ on the Tn*125-*like structure flanked by 5-bp TSD (AAGTT) mediated by IS*Aba125* (Fig. 1b). Two copies of IS*Aba14* transposons were also observed flanked by three possible TSD (TAT). The Tn*125-*like of DETAB-R21 has a 99.99% identity compared to the transposon in A. baumannii TP2 (GenBank accession CP060011), collected from clinical sample in USA in 2020 (Fig. 1b). Moreover, Tn*125-*like structure in A. baumannii ACN21 (GenBank accession CP038644), which is isolated from a blood sample in India in 2019, is also identical to that of DETAB-R21 with a percentage of 99.93%. In addition, a novel 4,343 bp IS*Aba1* composite transposon containing *bla*_CARB-16_ was also identified in the chromosome. Moreover, there was an amino acid mutation of *gyrA* (S81L), which could confer resistance to fluoroquinolones.

### Genetic analysis of plasmids contained in strain DETAB-R21.

We identified five plasmids in DETAB-R21, namely, pDETABR21-1 to pDETABR21-5, with sizes between 2,309-bp to 12,709-bp and GC contents ranging from 37% to 39%. None of the plasmids carried resistance genes (Table 1). Based on an analysis of the replicons, pDETABR21-4 and pDETABR21-5 carry the *repAci1 and repAci4* replication genes, respectively (Table 1). The remaining three plasmids contain so far undescribed replicons.

### Prophage regions in the chromosome.

**FIG 2:**
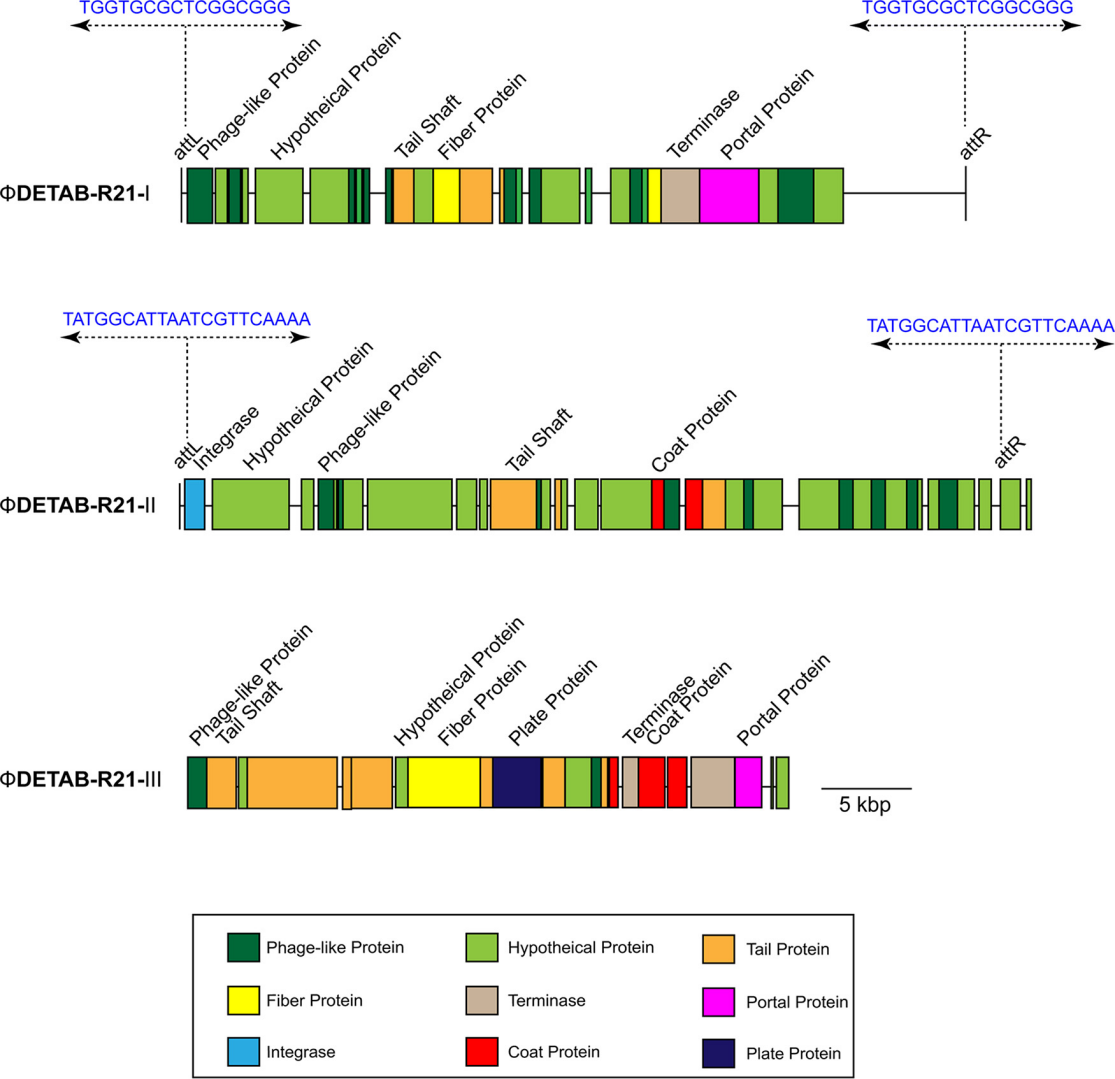
Predicted prophage regions within the A. baumannii DETAB-R21 chromosome. The length of ΦDETAB-R21-I, ΦDETAB-R21-II and ΦDETAB-R21-III are 37.4 kb, 51 kb and 24.2 kb, respectively. *att*L and *att*R are shown as blue bases at both ends of ΦDETAB-R21-I and ΦDETAB-R21-II. Genes are color-coded based on predicted functions.

Prophages play an important role in the biology of bacteria and can contribute to virulence of bacteria (12). Application of the tool PHASTER to predict the prophage regions, showed two prophages that are considered “intact” meaning they are likely able to undergo replication (ΦDETAB-R21-I, from 1,266,431-bp to 1,303,890-bp; ΦDETAB-R21-III, from 3,150,251-bp to 3,174,492-bp). In addition, one questionable prophage region was identified (ΦDETAB-R21-II, from 2,567,218-bp to 2,618,277-bp) which might potentially not be able to form phage progeny (Fig. 2). ΦDETAB-R21-I, ΦDETAB-R21-II and ΦDETAB-R21-III have a length of 37.4 kb, 51 kb and 24.2 kb with the GC content of 40.62%, 38.28% and 40.61%, respectively. Moreover, prophage ΦDETAB-R21-I and ΦDETAB-R21-II regions are both flanked by 16-bp and 21-bp attachment (*att*) sites, namely, *att*L and *att*R, respectively (Fig. 2). Based on the PHASTER tool, prophage ΦDETAB-R21-I was predicted to be intact due to the score of 110, whereas ΦDETAB-R21-II (score, 90) was classified as questionable while also flanked by the *att*L and *att*R sites. Although no *att*L and *att*R sites were found in ΦDETAB-R21-III, the putative prophage has a high score (150) and was identified as intact.

### *hmgA* plays a key role in pyomelanin production.

**FIG 3:**
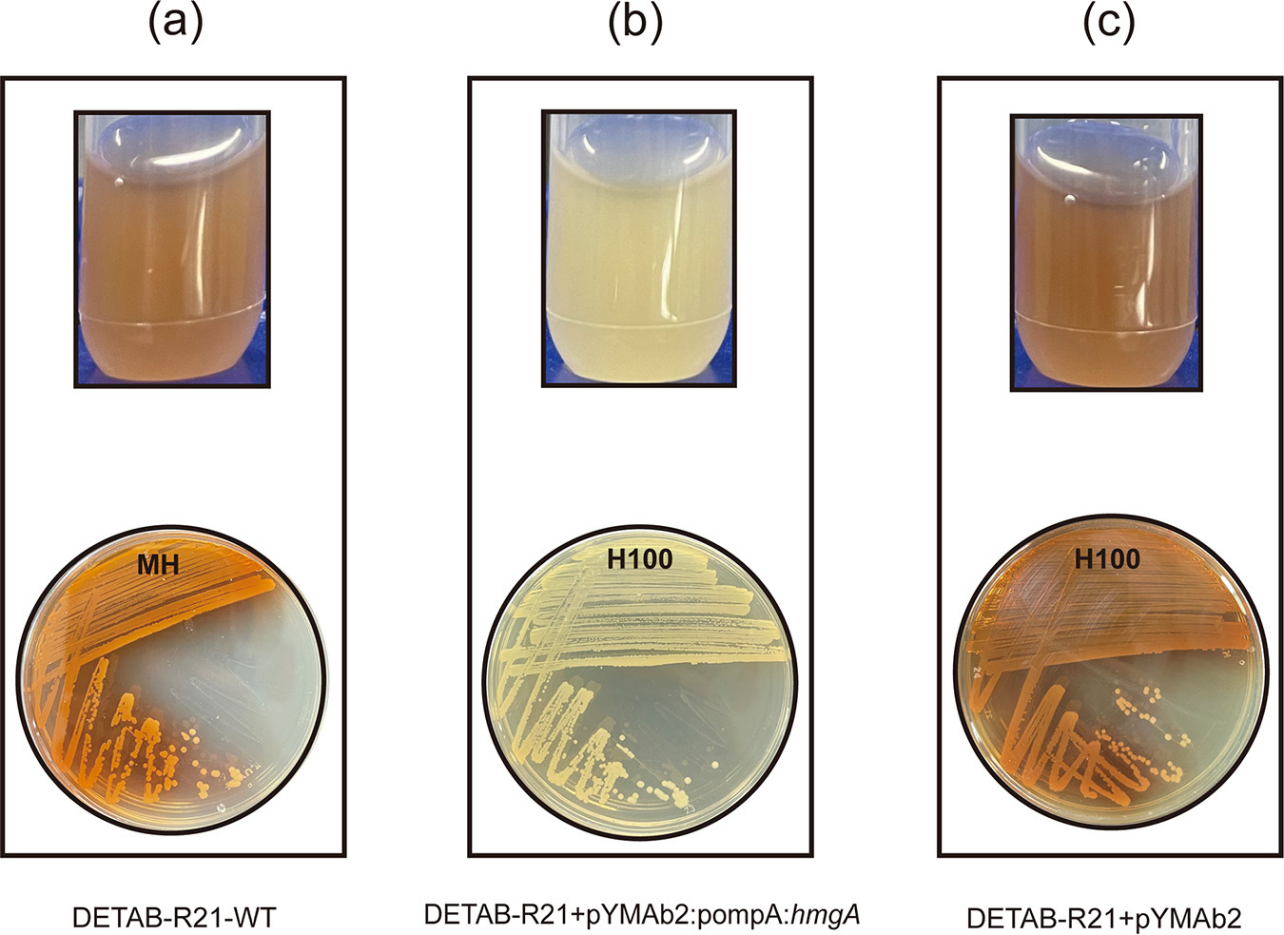
Pyomelanin generation of A. baumannii strains on MH agar plates and MH broth with or without hygromycin. (a) DETAB-R21-WT could produce pyomelanin on both media without hygromycin. (b) *hmgA* overexpression strains DETAB-R21+pYMAb2:pompA:*hmgA* failed to produce pigment. (c) DETAB-R21+pYMAb2 is an empty vector strain without hygromycin production in the presence of hygromycin.

The wild-type strain DETAB-R21 produced a pigment on MH agar plates, which we later identified as pyomelanin (Fig. 3). In contrast, XH1935 displays the usually observed phenotype with pale white colonies (data not shown). To explore the molecular basis of pigment production, we investigated the key genes in the pathway of pigment production. Results revealed that *hmgA* was interrupted by a Tn*2006* with 11-bp TSD (CATTTTTTT) (Fig. 1a). We then introduced a plasmid into the strain, leading to *hmgA* overexpression (DETAB-R21+pYMAb2:pompA:*hmgA*). Here, we observe that pigment production was abolished, leading to the normally observed phenotype (Fig. 3). The control, a complementation with a pYMAb2-Hyg^r^ empty vector, showed pigment production similar to the strain without any plasmid, producing pyomelanin at similar levels (Fig. 3).

### Antimicrobial agents and disinfectants susceptibility profiles.

The results of AST showed that DETAB-R21 is resistant to imipenem (128 mg/L), meropenem (256 mg/L), ceftazidime (>256 mg/L), levofloxacin (8 mg/L), and ciprofloxacin (8 mg/L) but still remained susceptible to gentamicin (2 mg/L), tobramycin (0.5 mg/L), colistin (0.125 mg/L), and tigecycline (2 mg/L) (Table 2). Moreover, DETAB-R21+pYMAb2:pompA:*hmgA*, DETAB-R21+pYMAb2 and XH1935 presented the same antimicrobial susceptibilities compared with DETAB-R21-WT. The MICs for three kinds of disinfectants showed that all stains have the same pattern of susceptibility.

**TABLE 2:** MICs of antibiotics against DETAB-R21

Isolates	Antibiotic^*a*^ minimum inhibitory concn (mg/L)^*b*^									Disinfectants^*c*^ (mg/L)		
	IMP	MEM	CAZ	GEN	TOB	LEV	CIP	COL	TGC	CHG	p-CP	Ca(OCl)2
DETAB-R21-WT	128 (R)	256 (R)	>256 (R)	2 (S)	0.5 (S)	8 (R)	8 (R)	0.125 (S)	2 (S)	4	256	>2048
DETAB-R21+ pYMAb2:pompA:*hmgA*	128 (R)	256 (R)	>256 (R)	2 (S)	1 (S)	8 (R)	8 (R)	0.125 (S)	2 (S)	4	256	>2048
DETAB-R21+pYMAb2	128 (R)	256 (R)	>256 (R)	2 (S)	1 (S)	8 (R)	8 (R)	0.125 (S)	2 (S)	4	256	>2048
XH1935	128 (R)	256 (R)	>256 (R)	2 (S)	1 (S)	8 (R)	8 (R)	0.125 (S)	2 (S)	4	256	>2048

a IMP = imipenem, MEM = meropenem, CAZ = ceftazidime, GEN = gentamicin, TOB = tobramycin, LEV = levofloxacin, CIP = ciprofloxacin, COL = colistin, TGC = tigecycline.

b Minimum inhibitory concentrations classed as resistant (R) or sensitive (S).

c CHG, chlorhexidine; p-CP, p-Chlorophenol; Ca(OCl)2, calcium hypochlorite.

### Growth ability and virulence comparison.

**FIG 4:**
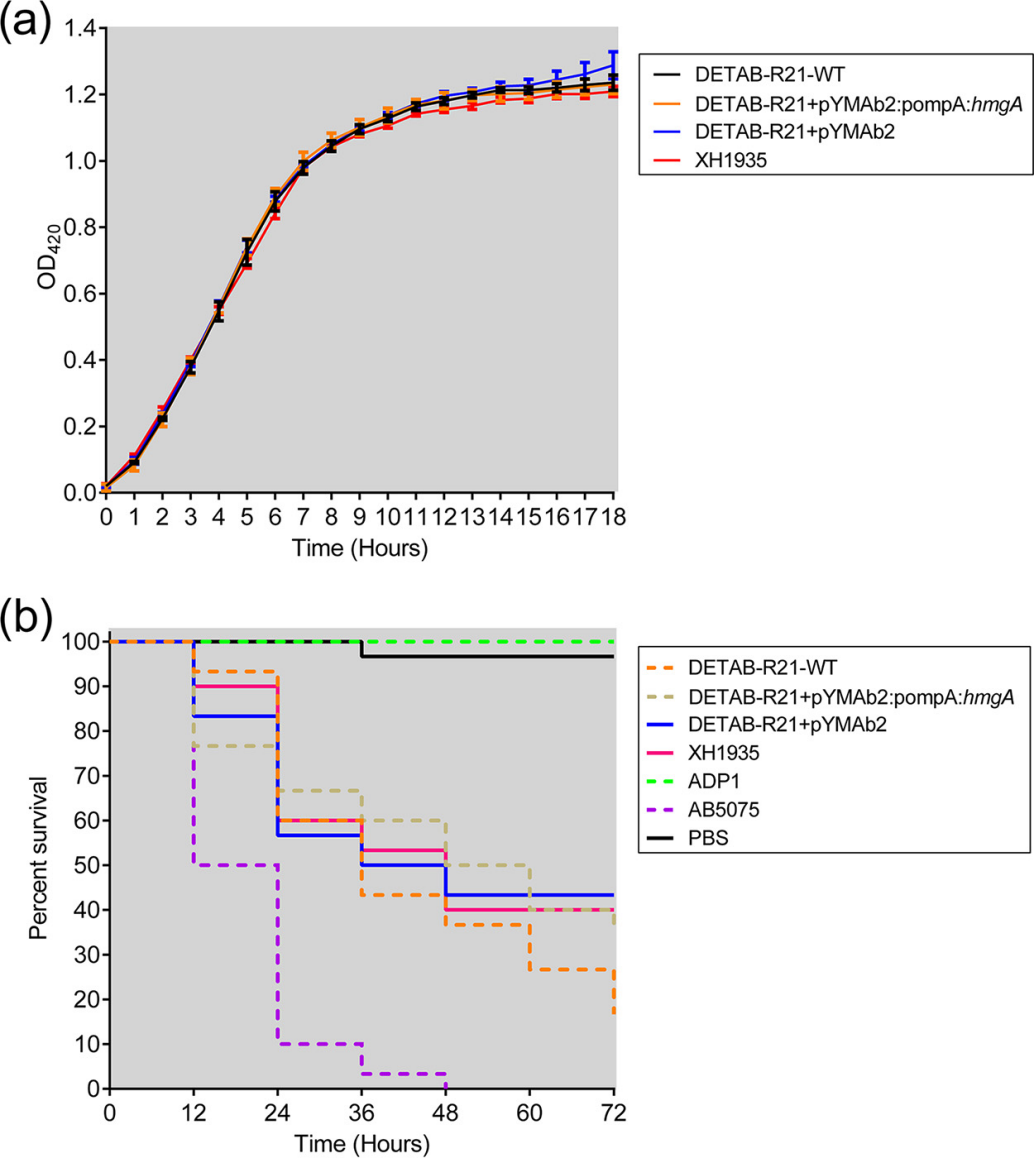
Growth curve and Kaplan-Meier survival curve. (a) cultures were incubated at 37°C with shaking and absorbance (OD420) were taken at the indicated time intervals. (b) Kaplan-Meier survival curve showing the virulence of different strains in G. mellonella. G. mellonella larvae (*n* = 30) were inoculated with 10^6^ CFU. Survival was recorded per 12 h for 3 days.

Growth dynamics were measured as a proxy for fitness in order to compare the bacterial propagation of strains that produce pyomelanin to those that do not. However, no observable differences were detected when comparing the wild type DETAB-R21, with or without plasmid-medicated *hmgA* overexpression (or the empty control vector) and strain XH1935 (Fig. 4a).

Next, we determined if pyomelanin influences the virulence of A. baumannii. To this end, the *in vivo* model G. mellonella was used. Half of the larvae died after 12 h postinjection in the case of the virulent strain A. baumannii AB5075 (Fig. 4b). All pyomelanin producing and no pyomelanin producing A. baumannii strains were less virulent compared to A. baumannii AB5075 (*P < *0.0001). More importantly, no differences were observed regarding the virulence of pyomelanin-producing DETAB-R21-WT and no pyomelanin producing strain XH1935 (*P = *0.1653). We also did not observe any statistical difference for DETAB-R21-WT and DETAB-R21+pYMAb2:pompA:*hmgA* (*P = *0.2011). The survival rates of DETAB-R21-WT, plasmid-complemented strains leading to *hmgA* overexpression or containing the empty control vector, and XH1935 strains were all highly similar to each other in the G. mellonella model (Fig. 4b).

### Bacterial survival rates under UV irradiation.

**FIG 5:**
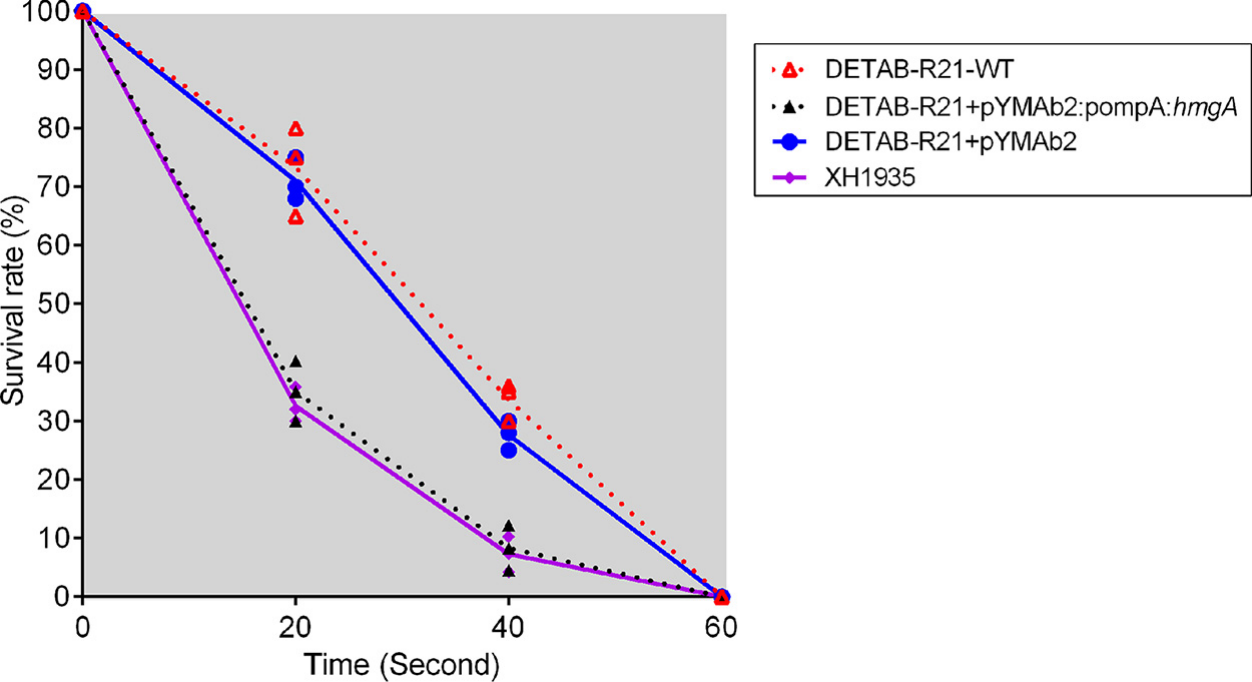
Bacterial survival rates under UV irradiation. Survival of DETAB-R21-WT, DETAB-R21+pYMAb2:pompA:*hmgA*, DETAB-R21+pYMAb2 and XH1935 after UV irradiation at 20 S, 40 S and 60 S, respectively. Survival was shown as data points.

As pigments have the ability to absorb light and protect light sensitive molecules such as DNA, we tested if the pigmented strain is less sensitive to UV radiation with three independent assays. Indeed, DETAB-R21-WT displayed a higher relative survival rate compared to XH1935 when exposed to 20 s (about 75% VS 35%) and 40 s (35% VS 10%) (Fig. 5). When pigment production was suppressed by introducing the wild-type gene on a plasmid, similar survival rates were observed in DETAB-R21+pYMAb2:pompA:*hmgA* and XH1935 (Fig. 5). All strains did not survive prolonged UV irradiation for 60 s or longer.

## DISCUSSION

Emergence and spread of multidrug resistant (MDR) A. baumannii are growing clinical problems throughout the world, posing a threat to public health (13, 14). This species has become one of the most important nosocomial pathogens that easily adapts to the hospital environment, particularly in the intensive care unit (ICU), and is able to disseminate rapidly in the environment and patients (15). Here, we have described the complete genome sequence of a rare pyomelanin-producing CRAB DETAB-R21, a ST164_Pas_ and ST1418_Oxf_ strain collected from an ICU patient. Previous reports have shown that resistance to carbapenems is usually mediated by OXA-23, OXA-24 and OXA-58 in A. baumannii (16). Coharboring of OXA-23 and NDM-1 are relatively rare, especially when embedded within the chromosome and not on plasmids. Moreover, many studies found that NDM-1-positive CRAB are ST85_Pas_ from the Middle East (2). Miltgen et al. described an outbreak of OXA-23 and NDM-1 positive ST1_Pas_ A. baumannii without special phenotype in the Southwest Indian Ocean Area of France (17). To our knowledge, our study is the first report of a ST164_Pas_ A. baumannii strain, producing pyomelanin, in which OXA-23 and NDM-1 occur at the same time.

The OCL and KL gene clusters, which are responsible for biosynthesis of outer core of lipooligosaccharide and capsule, are potentially useful epidemiological markers (18). A study from Kenyon et al. in Australia reported in 82 GC1 and GC2 A. baumannii strains mainly contain OCL1 and OCL3 (19). Another report described from Wyres et al. revealed that among 3029 A. baumannii genomes, the most common OCL types were OCL1 (2086, 68.87%) and OCL3 (272, 8.98 %) (18). Typing cell-surface polysaccharide-determining regions of the DETAB-R21 chromosome revealed that it contains the relatively rare OCL5. Bioinformatic analyses from other researchers identified the most common KL types to be KL2 (713 of 2948 genomes, 24.2%) (18), followed by KL9 (343, 11.6 %). DETAB-R21 again carries a less common type, the KL47 type.

Previous study found recombinase proteins XerC and XerD were responsible for resistance gene transfers via dif modules in plasmids, including carbapenemase genes *bla*_OXA-24_, (20) *bla*_OXA-58_ (21) and tetracycline resistance gene *tet*(39) (22). However, we were unable to observe their function of all XerC/XerD and XerD/XerC sites for the mobile of the resistance genes. Conversely, transposons play an important role in the dissemination of carbapenemase genes *bla*_OXA-23_ and *bla*_NDM-1_ in this study. Our previous research found the standard Tn*125* structure in a transferrable MDR plasmid in A. baumannii DETAB-P2 collected from the same ICU (21). A Tn*125*-like structure was also identified in this strain. Considering different TSD signature, we infer there are two possible reasons leading to the observed kind of Tn*125*-like structure formation. One possibility is mediated by two copies of IS*Aba125*, which differ from our previous report of Tn*125* (21). Another possibility is that *bla*_NDM-1_ was flanked by two copies of IS*Aba14* which then inserted into the bacterial chromosome. According to the Tn*125*-like structure analysis in A. baumannii DETAB-R21, it may have evolved from the A. baumannii isolate TP2 found in the USA in 2016 or from the A. baumannii strain ACN21 collected in India in 2018. While we previously identified prophages containing the resistance gene in A. baumannii strains (12), Thomas et al. also reported the possibility of the *bla*_NDM-1_ transfer by a prophage (23). In this study, three prophages regions were identified in the chromosome of strain DETAB-R21. However, whether these prophage regions are able to form phage particles able to transfer the resistance genes remains to be confirmed.

Another interesting finding is that a composite transposon Tn*2006* encoding the *bla*_OXA-23_ gene inserted into the chromosome and disrupted the *hmgA* gene, possibly leading to the production of dark brown pyomelanin. Our experimental data of *hmgA* overexpression strain construction established for the first time, that the *hmgA* gene plays a pivotal role in pyomelanin production. The impact of pyomelanin on phenotype in A. baumannii was further investigated. Certain virulence factors were identified in A. baumannii; Fonseca et al. considered that there might be a connection between pyomelanin production and virulence (10). However, in our study we tested virulence in the G. mellonella infection model, where we did not observe any difference in virulence whether or not the A. baumannii strains were producing pyomelanin. In addition, radiation resistance was observed in the pyomelanin-producing strain, meaning that the pigment may offer a potential protection effect for bacteria under UV. This could be of importance as UV sterilization is common but might be less effective in pigment producing strains.

Some limitations in this study remains, including the lack of knowledge of the biological functions of the dark brown pyomelanin. In addition, a murine infection model could be established to further evaluate the virulence level. Finally, either mechanism (natural transformation, conjugation, or phage transduction) of horizontal gene transfer (HGT) could transfer the Tn*2006* and Tn*125*-like composite transposons need further explored (24–26).

### Conclusions.

This study describes and confirms the function of the gene *hmgA* for the tyrosine metabolic pathway in a pyomelanin-producing CRAB, the first time. Medically more relevant is the description of the resistance genes in the strain, with three copies of OXA-23 and one copy of NDM-1 inserted into the chromosome via different composite transposons, demonstrating the strong plasticity of the genome in A. baumannii. Due to increasing numbers of highly resistant strains which show resistance to a plethora of antibiotics, effective monitoring and epidemic interventions should be implemented to decrease the dissemination of CRAB strains. If more pyomelanin-producing strains emerge, such as the one described in this study, this be a reason be concerned as the pigment may play a possible role in UV resistance.

## MATERIALS AND METHODS

### Ethics.

Ethical approval and informed consent were obtained from the Sir Run Run Shaw Hospital (SRRSH) local ethics committee, Zhejiang University (approval number 20190802-1).

### Patient information, bacterial isolation, medium, and culture condition.

One 83-year-old female patient was admitted with severe pancreatitis in the ICU of SRRSH, Hangzhou, China in 11.12.2020. The patient was treated with 3.0 g meropenem per day for 6 days and underwent screening every week. DETAB-R21 was collected from the oral swab in 19.01.2021. Specifically, the swab was put into 2 mL TSB with 0.1% sodium thiosulfate and incubated at 37°C for 24 h. 20 μL overnight culture was plated onto CHROMagar Acinetobacter sp. plates (CHROMagar, Paris, France) supplemented with 2 mg/L meropenem. After 24 h static incubation at 37°C, a single colony of presumptive A. baumannii was selected based on color and morphology, streaked onto a Mueller-Hinton (MH) agar plate (Oxoid, Hampshire, UK) and incubated overnight at 37°C. A single, isolated colony from the MH plate was selected and species were confirmed by MALDI-TOF MS (bioMérieux, Marcy-l’Étoile, France) and 16S rRNA gene sequencing. XH1935 was isolated from the sputum collected from another patient in the same ICU in 14.01.2021. The specific bacterial strains used in this study are listed in Table S1. The liquid medium used was MH broth (Oxoid, Hampshire, UK), which was supplemented with 100 hygromycin mg/L, as required.

### Whole-genome sequencing and sequence analysis.

Genomic DNA was extracted from A. baumannii DETAB-R21 and XH1935 using a Qiagen minikit (Qiagen, Hilden, Germany). Whole-genome sequencing was performed using both the Illumina HiSeq (Illumina, San Diego, USA) and the MinION (Nanopore, Oxford, UK) platforms (Weishu, Zhejiang, China). Assembly of the Illumina and Nanopore reads was performed using Unicycler v0.4.8 (27). Sequences were annotated using Prokka 1.14.0 (28). Multilocus sequence typing (MLST) with the Pasteur (29) and Oxford (30) schemes was performed via PubMLST (https://pubmlst.org/). Antimicrobial resistance genes were identified using ABRicate v0.8.13 (https://github.com/tseemann/abricate) with the ResFinder database (31) and insertion sequences were identified using ISFinder (32). Capsular polysaccharide (K locus) and lipoolygosaccharide (OC locus) were tested using Bautype (33) and *Kaptive* (18), respectively. XerC/XerD and XerD/XerC recombinases recognition sites were detected using p*dif*Finder (http://bacant.net/pdif/). The number of Single Nucleotide Polymorphisms (SNPs) in A. baumannii DETAB-R21 and XH1935 were calculated using Snippy v4.4.5 (https://github.com/tseemann/snippy) and snp-dists v0.6.3 (https://github.com/tseemann/snp-dists) (34). In addition, the PHAge Search Tool (PHASTER) was used for the prediction of bacteriophages (35).

### Construction of *hmgA* overexpression strain.

A complete fragment of *hmgA* was amplified from the A. baumannii strain XH1935. In addition, a strong promoter (from pompA) was also amplified from A. baumannii ATCC 17978. Primers are shown in Table S2. The products flanked by the recombination sequences were purified and cloned into the BamHI and SalI-digested shuttle plasmid vector pYMAb2-Hyg^r^ to yield the pYMAb2:pompA:*hmgA* using the ClonExpress II One Step Cloning kit (Vazyme Biotech Co., Ltd., Nanjing, China) according to the manufacturer’s recommendations. pYMAb2:pompA:*hmgA* was then introduced into A. baumannii DETAB-R21 wild type (DETAB-R21-WT) by electroporation. pYMAb2-Hyg^r^ vector alone was also transformed into DETAB-R21-WT as a empty control to yield DETAB-R21+pYMAb2.

### Antimicrobial and disinfectant agents susceptibility testing.

MICs for imipenem, meropenem, ceftazidime, gentamicin, tobramycin, ciprofloxacin, levofloxacin, colistin and tigecycline were determined using broth microdilution, with results interpreted using the CLSI 2021 guidelines. Escherichia coli ATCC 25922 served as the quality control strain. Moreover, MICs for disinfectants commonly used in the clinic setting, including chlorhexidine, p-Chlorophenol and calcium hypochlorite, were also measured using the broth microdilution method.

### Growth curve measurement.

Strains were streaked onto MH agar plates and incubated overnight at 37°C. Four independent cultures for every strain were grown overnight, diluted 1:1000 in MH broth and added into a flat-bottom honeycomb 100-well plate (Saue vald, Estonia) in three replicates. The plate was incubated at 37°C with agitation. Each culture was determined every 5 min for 18 h using a Bioscreen C MBR machine (Oy Growth Curves Ab Ltd., Finland) at a lower wide wavelength 420 nm as previous reports (36). Results were shown as OD_420_ value per hour with triplicate independent replications.

### Galleria mellonella infection model.

To compare the pathogenicity difference between pyomelanin and no pyomelanin producing strains, Galleria mellonella infection model was used as previously described (37, 38). Briefly, log-phase bacteria were centrifuged and resuspended in phosphate-buffered saline (PBS, Senrui, China) and underwent 10-fold serial dilutions to 1 × 10^8^ CFU/mL. Ten μL volume was injected into G. mellonella larvae (*n* = 10,0.2 to 0.3 g; Yuejiayin, Tianjin, China) using Hamilton syringe (Hamilton, USA). G. mellonella larvae were incubated at 37°C in the dark and survival was recorded every 12 h and monitored for 3 days. A. baumannii AB5075 was used as a virulence positive control, Acinetobacter baylyi ADP1 served as a nonpathogenic negative control. Experiments were conducted in triplicate.

### UV irradiation assay.

UV irradiation assay was carried out based on the description of Thoms and Wackernagel with minor modification (39). The UVC irradiation could damage or destruct the DNA/RNA in various microbes (e.g., bacteria, fungi, and viruses) (40, 41). Overnight cultures in LB broth were centrifuged and resuspended in PBS (Senrui, China) with a final density of 10^8^ cells/mL. Cells were irradiated under a UV lamp (Dose range: 0-6000 J/m2, Wavelength: 254 nm, Wattage: 30 W, G30T8 UV UVC, Osram, USA) with the 60 cm distance at 20, 40 and 60 s, respectively. The depth of cell suspension was < 1 mm. Cells was withdrawn and plated on MH agar plates after dilution for culture and further to calculate the bacterial survival rate with or without irradiation. Experiments were performed in triplicate.

### Statistical analysis.

G. mellonella survival rates were evaluated using Kaplan–Meier survival curves. Statistical significance was analyzed with a log-rank (Mantel–Cox) test using GraphPad Prism 8.0.2 (GraphPad Software, San Diego California USA). *P* values of < 0.05 was considered statistically significant.

### Data availability.

The complete sequences of the chromosome and five plasmids from A. baumannii DETAB-R21 have been deposited in the National Center for Biotechnology Information under BioProject PRJNA716893, BioSample SAMN23553142, genome accession numbers CP088895-CP088900. The A. baumannii XH1935 chromosomal sequence was deposited under BioProject PRJNA716893, BioSample SAMN23553143, genome accession number CP088894.
