# Supplementary material for: Description of a Rare Pyomelanin-Producing Carbapenem-Resistant Acinetobacter baumannii Strain Coharboring Chromosomal OXA-23 and NDM-1
Source: Microbiol Spectr. 2022 Aug 10;10(4):e02144-22. doi: 10.1128/spectrum.02144-22 (PMC9431530; doi:10.1128/spectrum.02144-22)
Supplement: Supplemental file 1 — Supplemental material. Download spectrum.02144-22-s0001.pdf, PDF file, 0.5 MB [file spectrum.02144-22-s0001.pdf]

	28 bp			
		6 bp central region		XerC/D recombinases recognition sites
1	GAATAAAATAA	TTATAT	TTATAATAAAT	C/D
2	CTTTTGCATAA	GTAGAA	ATATTTTATTT	D/C
3	TTTTTATATAA	AAATCT	TTAACTTAAAT	C/D
4	ATTAATGATAA	TAAGAA	TTATTTTTATT	D/C
5	AATTCCTTTAA	AATTTC	TCATTTTACTT	C/D
6	AGATAAGATAA	TGATTC	TTATTTACAAT	D/C
7	ATATTACATAA	TTAAAC	TTATATTTAAT	C/D
8	ACTTAACATAA	TATATA	TTATACGAACA	D/C
9	CTGTATTATAA	GTATTT	TTATGTGAAAT	C/D
10	AGTTTTTATAA	GACAAA	TCATGATAAAT	D/C
11	CTTTGGTATAA	TTAGCC	TAATATTTATT	C/D
12	AATACTGATAA	TCTATA	TTATAAGCAAT	D/C
13	CATTAGTTTAA	ACACGG	TTATCTGAATT	C/D
14	AAGTAATATAA	GTATTT	TTATTTTAAAT	D/C
15	TTTTAAAATAA	GCAGGC	TTATCTGCACT	C/D
16	GTTTCAGATAA	CACCCA	TTATGTTAAAT	D/C
17	AATTAACATAA	TACAGC	TTATGTGAAAG	C/D
18	GATTGATATAA	ACCGAC	TAAACTTAAAT	D/C
19	ACTCTATATAA	ATTTAG	TTATTCTAAAT	C/D
20	AATTGCCATAA	CGCCAC	TTAAACGAAGT	D/C

FIG S1. XerC/XerD recombinases recognition sites in *A. baumannii* DETAB-R21 chromosome. Recombinases recognition sites are labeled as C/D for the XerC-XerD orientation and D/C for XerD-XerC orientation. The length of C/D or D/C is 28 bp. It is comprised of two 11 bp half-sites, which are shaded as gray. Bases highlighted in different color in the center are 6 bp central region.

Table S1: Strains used in this study

Strains	Description	Source
A. baumannii DETAB-R21-WT[1]	Pyomelanin producing clinical strain with three copies of *bla*$_{OXA-23}$ and one copy *bla*$_{NDM-1}$, wild-type strain with *hmgA* gene disruption	This study
A. baumannii DETAB-R21+ pYMAb2:pompA:*hmgA*	Pyomelanin overexpressed strain	This study
A. baumannii DETAB-R21+pYMAb2	DETAB-R21-WT+pYMAb2-Hyg^{r}[2], as a control	This study
A. baumannii XH1935	5 SNPs[3] differences compared to WT, with complete *hmgA*, no pyomelanin producing clinical strain with two copies of *bla*$_{OXA-23}$ and one copy of *bla*$_{NDM-1}$	This study
A. baumannii AB5075	A pathogenic clinical isolate	(1)
A. baylyi ADP1	A non-pathogenic soil isolate	(2)
A. baumannii ATCC17978	A standard reference isolate	(3)

[1] WT, wild type
[2] Hyg, hygromycin; r, resistant
[3] SNP, single-nucleotide polymorphisms

Table S2: Oligonucleotide primers used in this study[1]

Primer name	Sequence (5'-3')
pompa--F	tgcggccgcaagctt**gtcgac**GTGTTATAGTGAGCTCAACTGTAGT
pompa--R	taatggcaaatgtcatGGATATCCTCCAGAGATAACAATTGTTG
hmgA-F	ctctggaggatatccATGACATTTGCCATTAAAAAAATACA
hmgA-R	cagcaaatgggtcgc**ggatcc**TTACGCGCCTAATTCTTCTTCA

[1] Lowercase letters indicate sequences for recombination. Recognition sequences for restriction enzymes are bold and underlined. F, forward (5') primer. R, reverse (3') primer.

References

1. Jacobs AC, Thompson MG, Black CC, Kessler JL, Clark LP, McQueary CN, Gancz HY, Corey BW, Moon JK, Si Y, Owen MT, Hallock JD, Kwak YI, Summers A, Li CZ, Rasko DA, Penwell WF, Honnold CL, Wise MC, Waterman PE, Lesho EP, Stewart RL, Actis LA, Palys TJ, Craft DW, Zurawski DV. 2014. AB5075, a Highly Virulent Isolate of Acinetobacter baumannii, as a Model Strain for the Evaluation of Pathogenesis and Antimicrobial Treatments. mBio 5:e01076-14.
2. Zhou H, Zhang L, Xu Q, Zhang L, Yu Y, Hua X. 2020. The mismatch repair system (mutS and mutL) in Acinetobacter baylyi ADP1. BMC Microbiol 20:40.
3. Mayer C, Muras A, Romero M, Lopez M, Tomas M, Otero A. 2018. Multiple Quorum Quenching Enzymes Are Active in the Nosocomial Pathogen Acinetobacter baumannii ATCC17978. Front Cell Infect Microbiol 8:310.
